# Health related quality of life in adolescents with idiopathic scoliosis: a cross-cultural comparison between two methods of treatment

**DOI:** 10.1186/1748-7161-7-S1-O6

**Published:** 2012-01-27

**Authors:** E D’agata, C Pérez-Testor, M Rigo, S Negrini, V Cigoli

**Affiliations:** 1 Universitat Ramon Llull, Barcelona, Spain; 2 Institut Elena Salvá, Barcelona, Spain; 3 ISICO, Milan, Italy; 4 Università Cattolica di Milano, Milan, Italy

## Purpose

The present study aims at evaluating the effects produced on HRQOL by two different methods of physiotherapy in adolescent population with Idiopathic Scoliosis (IS): *SEAS*, used in Milan (Italia) in ISICO center, and *Barcelona Scoliosis Physical Therapy School*, in E. Salvá Institut (Spain).

## Background

Studies related to HRQOL are generally few [1], controversial and besides there is a lack of research related to physiotherapy and HRQOL [2].

## Materials and methods

Twenty-one subjects took part to the study, ages ranging between 9 and 18 years. Thirteen of them were Italian (5 boys and 8 girls) and the 9 Spanish (2 boys and 7 girls). For all of them it was the first time to be visited. The materials used were: Rosenberg’s self-esteem test [3,4], Self Concept test [5], Body Satisfaction Scale [6] and SRS-22 questionnaire [7,8]. Tests were given three times: on the first visit, three and six months later.

## Results

Through a mixed ANCOVA, we found statistical differences between pre-test and post-test. In relation to the interaction effect, Time X Treatment, the Italian group improved in SRS-Pain while the E.Salvá group presented worse results at the end. However, the treatment had a significative effect on SRS-Self Image (p=0.016) and on Emotional Self Concept, as the E.Salvá group scored higher.

## Conclusions

Further researches will aim at increasing the sample size, in order to enrich the results, and at looking for more homogenous groups and centers (Country, setting, size, etc).
